# A quasi-experimental comparison of outcome-based hybrid learning and traditional instruction in environmental health education for preventive medicine undergraduates from Guilin Medical University

**DOI:** 10.3389/fpubh.2026.1887980

**Published:** 2026-08-06

**Authors:** Jiansheng Cai, Liang Cao, Huixia Zhang, Weiyi Pang, Yan Sun, Chengqiang Wang, Xi Zeng, You Li

**Affiliations:** 1 Department of Environmental Health and Occupational Medicine, School of Public Health, Guilin Medical University, Guilin, Guangxi, China; 2 The Guangxi Key Laboratory of Environmental Exposomics and Entire Lifecycle Heath, Guilin, Guangxi, China; 3 Department of Experimental Teaching Center, School of Public Health, Guilin Medical University, Guilin, Guangxi, China

**Keywords:** environmental health, outcome-based education, preventive medicine, teaching, undergraduate

## Abstract

**Introduction:**

In the post-pandemic era, online teaching has been widely adopted. Although traditional offline instruction remains essential for mastering medical knowledge, the integration of online and offline modalities under an outcome-based education (OBE) framework has gained an inevitable trend. However, the effectiveness of this hybrid model in environmental health education has not been fully evaluated. This study aims to evaluate the effectiveness of a hybrid teaching model grounded in OBE for the environmental health course.

**Methods:**

This study comprised the preventive medicine undergraduates from Guilin Medical University (the Classes of 2017–2020). A quasi-experimental design was employed, with the Class of 2017 serving as the control group (traditional face-to-face instruction) and the Classes of 2018–2020 as the experimental groups (OBE-based hybrid online-offline teaching). Educational efficacy was evaluated by comparing overall final exam scores, specifically analyzing written scores, objective item scores, and subjective item scores across groups. Surveys regarding hybrid teaching satisfaction and related indicators were conducted among the Class of 2018 majoring in preventive medicine.

**Results:**

The experimental group achieved markedly higher total scores relative to the control group (*Z* = −6.869, *p* < 0.001). The Classes of 2018–2020 outperformed the Class of 2017 in total scores (all *p* < 0.001); within the experimental group, the Class of 2020 scored significantly better than the Classes of 2018 and 2019 (both *p* < 0.001). The experimental group attained superior written examination scores (78.89 ± 8.15 vs. 68.61 ± 8.27, t = −11.140, *p* < 0.001) and median objective test scores (*p* < 0.01), with the Class of 2019 presenting the highest median objective score. Subjective scores also improved significantly in the experimental group and across successive classes (*p* < 0.001). The Cronbach’s alpha coefficient of the total scale was 0.911, with a coefficient of 0.961 for the satisfaction subscale. Students in the Class of 2018 reported high satisfaction (94.51%) with the OBE-based hybrid teaching model, and 40.66% of students preferred application-focused curriculum reform over theory-centered teaching.

**Conclusion:**

Integrating the OBE philosophy into hybrid online-offline teaching may potentially enhance teaching performance in environmental health course. Future studies could further optimize the design of online resources and the structure of offline interactions within hybrid learning.

## 1 Introduction

Preventive medicine is a discipline dedicated to exploring the interactions between external environmental factors and human health, characterized by strong practicality (1). In China, it is a formally recognized medical specialty within the national education framework. Undergraduate programs typically follow a 5-year structure, with the first 3 years focused on basic and clinical medicine, and the fourth year dedicated to core preventive disciplines such as environmental health, occupational health and occupational medicine, and social medicine (2). Environmental health, as a key sub-discipline, investigates how natural and residential environments influence human health and aims to establish health standards and preventive strategies (3). It integrates environmental science, toxicology, and epidemiology, emphasizing applied knowledge critical for training preventive medicine professionals (4, 5). Although environmental health is important to preventive medicine education, its instructional design and teaching methodologies remain exploratory, and a systematic teaching model has yet to be established.

The outcome-based education (OBE) model, introduced by William G. Spady in the 1980s (6), has gained global traction as an outcome-centered paradigm that prioritizes professional competencies through backward design, learner-centered instruction, and closed-loop quality assurance (7, 8). This approach has been successfully implemented in engineering and is now expanding into medical education. In parallel, web-based e-learning has become integral to modern medical training, especially after the 2020 pandemic, when offline instruction was suspended worldwide (9). However, traditional online methods such as video lectures have shown limited effectiveness in boosting engagement or achieving learning goals (10, 11). In the post-pandemic era, there is an urgent demand for a hybrid teaching model that effectively integrates online and offline elements (12, 13). This hybrid model enhances flexibility, promotes self-directed learning, and improves teaching efficiency by expanding access to content and enabling real-time feedback (14, 15).

While prior studies have explored hybrid teaching innovations in medical education—such as the integration of Small Private Online Courses (SPOCs) with problem-based learning (PBL) in Neurology (16)—no existing literature has documented the application of an SPOC-based, OBE-oriented hybrid teaching model in environmental health. Guided by the OBE framework, this study implements a fully integrated online-offline hybrid teaching model using the SPOC platform (17). By comparing the academic performance of preventive medicine undergraduates receiving traditional offline instruction with those in the hybrid model, this research aims to enhance learning motivation, foster intellectual curiosity, and promote the integration of theoretical knowledge with practical application. Focusing on the Classes of 2018–2020 at Guilin Medical University—successive groups that represent a natural progression in curriculum reform—this study enables longitudinal evaluation of learning outcomes. Environmental health plays a critical role in public health preparedness and is directly relevant to pressing issues such as food safety and environmental exposure. However, a significant pedagogical gap exists in this field. To address this gap, this study provides a systematic, evidence-based framework for training high-quality, application-oriented medical talents. Specifically, the research objectives were formulated to conduct a quasi-experimental comparison between outcome-based hybrid learning and traditional instruction in environmental health education. This comparison was carried out among preventive medicine undergraduates at Guilin Medical University.

## 2 Methods

### 2.1 Study design

This was a non-randomized controlled trial (NRCT) with a parallel-group, historical control design. The study aimed to evaluate the effectiveness of an online-offline hybrid teaching model (Rain Classroom + SPOC) guided by OBE in environmental health education.

### 2.2 Study subjects

Full-time undergraduate students majoring in preventive medicine (enrolled in the Classes of 2017–2020) at Guilin Medical University were recruited as study subjects from March 2020 to July 2024. All participants were fourth-year students who had completed both basic and clinical medicine courses. A total of 402 students were included: 107 in the control group (the Class of 2017) and 295 in the experimental groups (the Classes of 2018–2020).

### 2.3 Intervention details

#### 2.3.1 Allocation of interventions

Unit of delivery: Teaching was delivered to intact the entire classes as independent units.

Deliverer: All teaching activities for both groups were undertaken by full-time faculty specializing in environmental health.

Participants were not randomly assigned. Group allocation was determined by their academic year using a historical control design.

The Class of 2017 (enrolled before the full implementation of the hybrid teaching model) was designated as the control group, as they received conventional teaching without structured hybrid intervention. The Classes of 2018–2020 were assigned to the experimental group, as they were exposed to the full hybrid teaching model after curriculum reform. No allocation sequence was generated due to the non-random, academic year-based assignment.

#### 2.3.2 Guiding principles

Both groups followed the same course syllabus and learning objectives, but the experimental group adopted a student-centered, OBE-guided online-offline hybrid teaching model, while the control group received conventional instruction.

Setting: Offline classroom sessions were completed in on-campus multimedia teaching rooms. All pre-class and post-class online learning tasks were finished remotely by students in their dormitories or personal residences via mobile phones or laptops.

#### 2.3.3 Control group intervention

Instructional mode: Conventional classroom teaching supplemented with limited online resources (developed during the 2020 COVID-19 pandemic).

Online components: A small portion of pre-class instruction was delivered via live streaming (Tencent Meeting/QQ Live) and SPOC video resources, but no structured online assessments or interactive activities were implemented.

Offline components: Multimedia-based lectures introduced through clinical cases, with use of Rain Classroom (18) or participatory teaching methods.

#### 2.3.4 Experimental group intervention

Integrated model: “Rain Classroom+SPOC” hybrid teaching with complete online-offline integration and mandatory online assessments. For a thorough introduction to SPOC, consult the cited academic literature (19).

Pre-class (online): Pre-class assignments and SPOC video materials (Environmental Health-specific) were distributed via Rain Classroom; students completed SPOC learning and preliminary quizzes independently.

In-class (offline): Heuristic and discussion-based teaching was conducted using multimedia and clinical cases. Rain Classroom facilitated real-time interaction (e.g., in-class quizzes, anonymous voting) to assess immediate learning outcomes. Instructors provided targeted explanations based on students’ pre-class preview data (Rain Classroom) and SPOC learning/test results.

Post-class (online): Students completed structured online assessments (e.g., unit tests, phased assessments) and participated in online discussions via the SPOC platform.

Intervention integrity: All instructors received standardized training on hybrid teaching methodologies and Rain Classroom/SPOC operation to ensure consistent delivery. Uniform textbooks, syllabi, and teaching schedules were adopted across groups.

Exposure quantity and duration: The full Environmental Health course spanned 18 teaching weeks with 5 in-class hours per week. Students were assigned one SPOC learning module and corresponding pre-class quiz each week, with an expected self-learning duration of 30–40 min per module. A total of 8 staged online unit assessments were arranged throughout the semester. Each offline case-based discussion session lasted 120 min.

Overall time span: The complete set of hybrid teaching interventions for each class was implemented over one full academic semester (18 weeks).

Adherence promotion strategies: Completion of SPOC videos, pre-class quizzes and post-class online assessments accounted for certain proportion of students’ formative process scores. Automated reminder notifications were pushed via Rain Classroom 24 h before each online task deadline, and the instructors provided targeted tutoring for students with low task completion rates.

### 2.4 Research hypothesis

It is hypothesized that compared with students receiving conventional offline teaching, undergraduates taught via the outcome-based education integrated online-offline hybrid model will achieve significantly higher overall course scores, written examination scores, objective question scores and subjective question scores for the Environmental Health course. Additionally, students from the Class of 2018 will report high satisfaction with the OBE-based hybrid teaching design.

### 2.5 Outcome measures

#### 2.5.1 Primary outcome

Course performance (total scores), measured by the final composite grade (100 points total).

Summative assessment (written exam scores): Standardized written test covering all course content (identical for both groups).

Objective and subjective assessment (objective test and subjective test scores): The written examination consisted of two parts: objective questions and subjective questions. The objective questions assessed students’ memorization and recall abilities, while the subjective questions evaluated their comprehension and comprehensive analytical skills.

All grades were jointly evaluated by instructors teaching the environmental health course. The assessment covered all content delivered in the course, and the question types and assessment requirements complied with the university’s official examination standards. The question types, number of items and score distribution remained identical every year, and the teaching staff and grading criteria were consistent across all classes, which helped reduce assessment bias.

#### 2.5.2 Secondary outcome

Student satisfaction with teaching and reform suggestions (20), measured via a self-designed structured questionnaire (the Class of 2018 only, as a representative subset of the experimental group). The Class of 2018 was the first group to receive hybrid teaching. Subsequent follow-up research could only be sustainably carried out if this class showed satisfactory feedback. The survey results verified their high satisfaction level, so we only conducted satisfaction-related questionnaires among the Class of 2018.

Measurement tool: Electronic questionnaire distributed via Questionnaire Star (https://www.wjx.cn/) (21).

Survey content: Satisfaction with hybrid teaching engagement (e.g., pre-class preview, in-class interaction, online assessments) and feedback on curriculum reform priorities.

### 2.6 Blinding

Blinding of both student participants and instructors was not feasible. Students were able to clearly distinguish between conventional and hybrid teaching modalities, and instructors were required to deliver their allocated teaching interventions, meaning blinding could not be implemented for either group. Blinding of outcome assessors was successfully implemented. All exam papers were scanned into the computer, and the teachers grade the scanned electronic versions. Each teacher was assigned three to four subjective questions for marking. During the grading process, students’ names and student ID numbers were not displayed; only the question numbers were shown. All teachers grade the papers according to the scoring criteria, and after grading was completed, the course coordinator conducted sample checks. If any issues were identified, two teachers would jointly revise the scores to ensure the authenticity and anonymity of the grades. The questionnaire data were fully de-identified prior to analysis to mitigate assessment bias.

### 2.7 Statistical analysis

Statistical analyses were conducted with SPSS version 28.0 (IBM Corp., United States). For normally distributed data, independent two-sample t-test was applied to compare differences between two groups, and one-way analysis of variance (ANOVA) was used to detect intragroup differences, with post-hoc pairwise comparisons conducted via the least significant difference (LSD) test. Skewed continuous data were presented as median (M) and interquartile range (P_25_, P_75_). The Wilcoxon rank-sum test was adopted for two-group comparison, and the Kruskal-Wallis H test was used for intragroup comparisons. A two-tailed *p* < 0.05 was considered statistically significant.

Missing data: No missing data was reported for course performance (all students completed the final examination). A total of 91 valid questionnaires were collected (response validity rate: 97.85%), so no imputation was needed.

### 2.8 Quality control and bias mitigation

Standardization of teaching materials and syllabi: Uniformly designated textbooks were adopted. The teaching schedule, key and difficult points, as well as instructional objectives, were strictly implemented in accordance with the approved syllabus to ensure consistency of course content.

Standardization of classroom instruction: All instructors received standardized training in hybrid teaching methodologies to maintain the quality and coherence of classroom delivery.

Prior to the formal survey: The questionnaire was validated by experts specializing in environmental health. A small-scale pilot survey was implemented, and revisions of the questionnaire were performed according to feedback obtained from the pilot investigation.

Anonymous survey administration: An anonymous questionnaire design was employed to encourage candid feedback from students. Logical consistency checks and mandatory item validation were performed during the data collection.

Data handling: Data were extracted directly from the Questionnaire Star platform into Excel files for subsequent analysis, minimizing errors from manual data entry.

## 3 Results

### 3.1 Comparison of total scores in the environmental health course among different groups

As total scores deviated from normality, non-parametric analyses were employed. The total scores in the environmental health course, expressed as median (IQR), were 80.00 (77.00, 84.00) for the control group and 85.00 (81.00, 88.00) for the experimental group. A statistically significant difference in total scores was found between the two groups (*Z* = −6.869, *p* < 0.001) (Table 1).

**Table 1:** Comparisons of total scores and objective test scores of environmental health for different groups with the Wilcoxon rank-sum test [M (P_25_, P_75_)].

Group	*n*	Total scores	Objective test scores
The control group	107	80.00 (77.00, 84.00)	38.00 (36.00, 40.00)
The experimental group	295	85.00 (81.00, 88.00)	40.00 (36.00, 42.00)
Total	402	84.00 (79.75, 87.00)	39.00 (36.00, 42.00)
*Z*		−6.869	−2.683
*P*		<0.001	0.007

M = median; P_25_ = 25th percentile; P_75_ = 75th percentile.

The results of the subgroup analysis were shown as follows: Class of 2018: 83.00 (80.00, 86.00); Class of 2019: 83.00 (80.00, 87.00); Class of 2020: 87.00 (84.25, 90.75). The Kruskal-Wallis H test revealed a statistically significant difference in scores among the four classes (*χ^2^* = 83.739, *p* < 0.001). Post-hoc pairwise comparisons indicated that the total scores for the Classes of 2018, 2019, and 2020 were all significantly higher than that of control group (all *p* < 0.001). Additionally, the scores of the Class of 2020 were significantly higher than those of the Class of 2018 and the Class of 2019 (both *p* < 0.001) (Table 2).

**Table 2:** Comparisons of total scores and objective test scores in environmental health for different classes with the Kruskal-Wallis H test [M (P_25_, P_75_)].

Class	*n*	Total scores	Objective test scores
2017	107	80.00 (77.00, 84.00)	38.00 (36.00, 40.00)^&^
2018	93	83.00 (80.00, 86.00)^*#^	38.00 (35.00, 42.00)^&^
2019	90	83.00 (80.00, 87.00)^*#^	41.50 (39.00, 44.00)
2020	112	87.00 (84.25, 90.75)^*^	39.00 (36.00, 41.00)^&^
Total	402	84.00 (79.75, 87.00)	39.00 (36.00, 42.00)
*χ^2^*		83.739	28.713
*P*		<0.001	<0.001

M = median; P_25_ = 25th percentile; P_75_ = 75th percentile. Post-hoc pairwise comparisons showed that: ① ^*^: scores of the Classes of 2018, 2019, and 2020 were significantly higher than that of the Class of 2017 (all *p* < 0.001); ② ^#^: score of the Class 2020 was significantly higher than those of the Classes of 2018 and 2019 (both P<0.001). Results of the post-hoc LSD test indicated: *p* < 00.01; ③ ^&^: for comparisons of the Classes of 2017, 2018, and 2020 vs. the Class of 2019, all *p* < 00.001.

### 3.2 Comparison of written examination scores in the environmental health course among different groups

Since the written examination scores followed a normal distribution (both *P*>0.05), independent two-sample t test and ANOVA were conducted to compare written mean scores. Mean written examination scores were 68.61 ± 8.27 in the control group and 78.89 ± 8.15 in the experimental group, respectively, with a statistically significant intergroup difference (*t* = −11.140, *p* < 0.001) (Table 3).

**Table 3:** Comparisons of written examination scores and subjective test scores in environmental health for different groups with t test ( $\bar{\chi }$ ± s).

Group	*n*	Written examination scores	Subjective test scores
The control group	107	68.61 ± 8.27	30.79 ± 5.74
The experimental group	295	78.89 ± 8.15	39.75 ± 5.41
Total	402	76.15 ± 9.35	37.37 ± 6.78
*|t|*		11.140	14.452
*P*		<0.001	<0.001

One-way ANOVA revealed significant score differences among the 2017, 2018, 2019 and 2020 Classes (*F* = 42.247, *p* < 0.001), whose respective mean scores were 68.61 ± 8.27, 78.49 ± 7.78, 80.02 ± 7.93, and 78.31 ± 8.58. Post-hoc pairwise comparisons confirmed that the 2018, 2019 and 2020 Classes all obtained substantially higher scores than the 2017 Class (all *p* < 0.001) (Table 4).

**Table 4:** Comparisons of written examination scores and subjective test scores in environmental health for different classes with one-way ANOVA ( $\bar{\chi }$ ± s).

Class	*n*	Written examination scores	Subjective test scores
2017	107	68.61 ± 8.27	30.79 ± 5.74
2018	93	78.49 ± 7.78^*^	40.30 ± 4.70^*^
2019	90	80.02 ± 7.93^*^	39.17 ± 5.62^*^
2020	112	78.31 ± 8.58^*^	39.78 ± 5.78^*^
Total	402	76.15 ± 9.35	37.37 ± 6.78
*F*		42.247	70.254
*P*		<0.001	<0.001

* *p* < 0.01 for comparisons of the Class of 2017 vs. the Classes of 2018, 2019, and 2020 (all *p* < 0.001).

### 3.3 Comparison of objective test scores in the environmental health course among different groups

As objective test scores were non-normally distributed, non-parametric analyses were applied. Median objective test scores were significantly higher in the experimental group 40.00 (36.00, 42.00) than in the control group 38.00 (36.00, 40.00) (*p* < 0.01) (Table 1).

The median objective test score for the Class of 2017 was 38.00 (36.00, 40.00). Corresponding scores for the Classes of 2018, 2019, and 2020 were 38.00 (35.00, 42.00), 41.50 (39.00, 44.00), and 39.00 (36.00, 41.00), respectively. A Kruskal– Wallis H test identified a statistically significant difference in median scores across the four classes (*χ^2^* = 28.713, *p* < 0.001). Post-hoc pairwise comparisons with Dunn’s test showed that the median score for the Class of 2019 was significantly higher than those for the Classes of 2017, 2018, and 2020 (all *p* < 0.001) (Table 2).

### 3.4 Comparison of subjective test scores in the environmental health course among different groups

Given that scores on subjective questions adhered to a normal distribution, intergroup comparisons of mean scores were performed using *t* test and ANOVA. The mean subjective test score of the experimental group was higher than that of the control group, and the difference was statistically significant (Table 3).

The mean subjective test score for the Class of 2017 was 30.79 ± 5.74. For the Classes of 2018, 2019, and 2020, the mean scores were 40.30 ± 4.70, 39.17 ± 5.62, and 39.78 ± 5.78, respectively. A one-way analysis of variance (ANOVA) showed a statistically significant difference in scores across the four classes (*F* = 70.254, *p* < 0.001). Post-hoc pairwise comparisons indicated that the mean scores for the Classes of 2018, 2019, and 2020 were all significantly higher than that of the Class of 2017 (all *p* < 0.001) (Table 4).

### 3.5 Reliability analysis of the questionnaire

The Cronbach’s alpha coefficient of the total scale was 0.911, with a coefficient of 0.961 for the satisfaction subscale. This coefficient was more than 0.7, indicating that the questionnaire could be used for subsequent research.

### 3.6 Evaluation of teaching satisfaction for students in the environmental health course

The students expressed a generally high overall satisfaction with the hybrid online and offline teaching model based on the OBE philosophy. Across all items, the highest proportion of dissatisfaction was 5.49%, as presented in Table 5.

**Table 5:** Evaluation of students’ teaching effect on environmental health *n* (%) (*n* = 91).

Items	Very satisfied	Satisfied	Averagely satisfied	Not at all satisfied
The instructional model that combines SPOC Classroom and Rain Classroom	21 (23.08)	48 (52.75)	17 (18.68)	5 (5.49)
The teaching content and instructional hours distributed between SPOC Classroom and Rain Classroom	20 (21.98)	43 (47.25)	25 (27.47)	3 (3.30)
The classroom climate	23 (25.27)	57 (62.64)	10 (10.99)	1 (1.10)
The academic workload	16 (17.58)	51 (56.04)	19 (20.88)	5 (5.49)
The assessment system	20 (21.98)	50 (54.95)	20 (21.98)	1 (1.10)
The instructors’ guidance approaches	21 (23.08)	59 (64.84)	11 (12.09)	0 (0.0)
Your own autonomous learning competence	17 (18.68)	46 (50.55)	23 (25.27)	5 (5.49)
Your in-class participation and interaction	15 (16.48)	59 (64.84)	15 (16.48)	2 (2.20)

### 3.7 The impact of hybrid teaching on students’ learning

A total of 80.22% of participating students held the view that hybrid teaching contributes to effective learning in environmental health course.

### 3.8 The focus of teaching reform in environmental health

Regarding the key focus of teaching reform for environmental health course, students identified a shift in teaching content from theory-oriented to application- oriented as the most important aspect, accounting for 40.66%. This was followed by the transformation of teaching methods from traditional to modern approaches (26.37%), while other aspects represented the smallest proportion (5.49%), as shown in Table 6.

**Table 6:** Key content of the environmental health teaching reform as perceived by students (*n* = 91).

Item	*n* (%)
Change in teaching methods, shift from teacher-centered to student- centered	17 (18.68)
Change in teaching means: transition from traditional to modern teaching means	24 (26.37)
The shift of teaching content from basic theories to cutting-edge advances in this discipline	37 (40.66)
Change in teaching evaluation methods: shift from outcome- dominated evaluation to a combination of outcome-based and process-based evaluation	8 (8.79)
Other	5 (5.49)

## 4 Discussion

The implementation of an OBE-oriented online-offline hybrid learning model in environmental health education had yielded significant improvements in student learning outcomes. Compared to the traditional teaching approach used in the Class of 2017, the hybrid model—applied progressively across the Classes of 2018–2020—consistently produced higher academic performance, with statistically significant gains in final total scores (*p* < 0.001) and written test results. Notably, the Class of 2020 achieved the highest median score (85.00), reflecting a clear year-on-year enhancement in performance. These findings aligned with the core principles of OBE, particularly the cycle of continuous improvement through iterative feedback and curriculum refinement (22). This progression mirrored the OBE ideal of aligning teaching practices with measurable learning outcomes and adapting instruction based on evidence—thus transforming education from a static delivery model into a dynamic, responsive system. The results were further supported by recent meta-analyses indicating that hybrid learning in health professions education consistently outperforms traditional instruction in promoting knowledge acquisition, engagement, and competency development (23, 24).

Further analysis of subjective-question performance provided additional evidence of the advantages of hybrid learning. The hybrid learning groups achieved significantly higher subjective-question scores compared to the traditional teaching group (all *p* < 0.001). Subjective questions primarily assess knowledge application and innovative thinking; the marked improvement in these scores reflects how hybrid learning-through the integration of online self-directed inquiry and offline interactive discussion-effectively overcomes the unidirectional knowledge transmission typical of traditional teaching. Prior studies have shown that combining OBE principles with technology-enhanced instruction strengthens the development of students’ comprehensive competencies (25). The “online-offline” synergy established in hybrid learning provides a clear pathway for translating knowledge into practical ability (26), a conclusion corroborated by the improved subjective-question performance observed in the present study.

Notably, objective-question scores revealed a distinct pattern. The Class of 2019 achieved the highest objective-question score (41.50), significantly exceeding that of other classes (*p* < 0.001). In contrast, the Class of 2020-despite obtaining the best overall performance did not rank highest in objective questions (39.00). Objective questions primarily assess retention of foundational knowledge, an area in which the structured, lecture-based format of traditional teaching holds inherent advantages. Conversely, the more fragmented nature of hybrid learning may lead to less systematic consolidation of basic knowledge (27). The strong objective-question performance of the Class of 2019 may be attributed to greater engagement with objective practice exercises and more thorough textbook review. This implies that hybrid learning should aim to achieve a balance between imparting foundational knowledge and cultivating comprehensive competencies — a view supported by the findings of Forde et al. (27) in their research on practical skill instruction.

More fundamentally, these performance differences arise from core disparities between the two instructional paradigms. Traditional teaching adopts an instructor-centered model, which renders it challenging to address students’ personalized learning needs. By contrast, hybrid learning, rooted in OBE principles, establishes a complete “input-internalization-output” learning cycle (24). The online learning component enables students to progress at their own pace and revisit academically challenging content, whereas the offline segment centers on interactive discussion and hands-on applied practice. This instructional model harnesses faculty guidance while prioritizing learner agency, thereby accommodating students with varied levels of academic proficiency. Existing research confirms that the advantages of hybrid learning in enhancing learning motivation and academic performance stem from its emphasis on student autonomy (23). The observed improvements in overall scores in the present study further corroborate the efficacy of this pedagogical strategy.

The overall student satisfaction rate with hybrid teaching reached 94.51%, which was consistent with the findings of Feng X (28), who stated that the hybrid PBL approach was associated with higher student satisfaction in the obstetrics and gynecology nursing course. These showed that the majority of students preferred hybrid teaching compared with offline teaching. Furthermore, 80.22% of survey participants affirmed that hybrid teaching facilitated the learning of environmental health. In line with outcomes observed in pediatric dentistry courses adopting a hybrid learning environment (29), this evidence demonstrated that hybrid teaching successfully fostered students’ learning motivation in medical disciplines. Meanwhile, the participants stated that the focus of teaching reform in environmental health should be shifting from a theory-oriented model to an application-oriented one. This was closely associated with the post competencies required for preventive medicine majors (30).

Overall, hybrid teaching underpinned by OBE effectively boosts students’ learning engagement and academic performance. It is therefore worthy of continuous refinement to promote teaching reform in environmental health and keep pace with the evolving needs of the AI era.

The findings of this study provide empirical support for OBE-driven teaching reform. However, certain limitations should be acknowledged. First, potential confounding factors such as differences in students’ baseline academic ability were not controlled. Second, Extending the satisfaction survey to other grades would enhance the comparability of the findings. Third, the study was conducted at a single institution, which may limit the generalization of the findings to other contexts. Moreover, expanding the sample size across multiple institutions, conducting longitudinal follow-up, and incorporating measures of deep learning engagement (31) will offer a more comprehensive basis for the ongoing improvement of teaching models. This aligns with the consensus in hybrid learning research that “long-term effects deserve prioritized attention” (23, 32).

## 5 Conclusion

Integrating the OBE philosophy into hybrid online-offline teaching may potentially enhance teaching performance in environmental health course. Future studies could further optimize the design of online resources and the structure of offline interactions within hybrid learning.

## Data Availability

The original contributions presented in the study are included in the article/supplementary material, further inquiries can be directed to the corresponding author.
